# LAPAROSCOPIC LIVER RESECTION FOR BENIGN TUMORS: THE CURRENT POSITION

**DOI:** 10.1590/0102-672020210002e1641

**Published:** 2022-01-31

**Authors:** Paulo HERMAN, Gilton Marques FONSECA, Jaime Arthur Pirola KRUGER, Vagner Birk JEISMANN, Fabricio Ferreira COELHO

**Affiliations:** 1 Unidade de Cirurgia Hepática, Hospital de Clínicas, Departmento of Gastroenterologia, Faculdade de Medicina da Universidade of São Paulo - USP - Sao Paulo, Brasil.

**Keywords:** Laparoscopy, Adenoma, Liver Cell, Cystadenoma, Focal nodular hyperplasia, Angiomyolipoma, Hemangioma, Laparoscopia, Adenoma de células hepáticas, Cistadenoma, Hiperplasia nodular focal do Fígado, Angiomiolipoma, Hemangioma

## Abstract

The main indications of the use of laparoscopic liver surgery (LLS), in the early days, were benign liver lesions. As LLS became more popular, indications for malignant diseases outnumbered those for benign ones. This study aims to rule out the indications and results of LLS for the treatment of benign liver tumors.

**METHODS:**

Out of 445 LLS performed in a single center, 100 (22.4%) were for benign tumors. The authors discuss the indications for resection and present their perioperative results.

**RESULTS:**

In total, 100 patients with benign tumors were evaluated. Specifically, these were as follows: 66 cases of hepatocellular adenomas; 14 cases of biliary mucinous neoplasm; 13 cases of focal nodular hyperplasia; 4 cases of angiomyolipomas; and 3 cases of hemangiomas with a mean size of 7.6 cm (ranging from 3.1 to 19.6 cm). The total morbidity rate was 19%, with 9% classified as Clavien-Dindo grades 3 or 4. No mortality was observed.

**CONCLUSION:**

LLS for benign liver tumors is safe and presents excellent results. However, indications for resection are increasingly restricted and should not be performed just because it is a minimally invasive procedure.

## RESUMO - RACIONAL

As principais indicações das hepatectomias video-laparoscópicas (HVL), inicialmente, eram nas lesões hepáticas benignas. À medida que a HVL se tornou mais popular, as indicações de doenças malignas superaram as de doenças benignas. Este estudo teve como objetivo discutir as indicações e resultados da HVL para o tratamento de tumores hepáticos benignos.

**MÉTODOS:**

De 445 HVL realizadas em um único centro, 100 (22,4%) foram para tumores benignos. Os autores discutem as indicações para ressecção e apresentam seus resultados perioperatórios.

**RESULTADOS:**

No total, 100 pacientes com tumores benignos foram avaliados, a saber: 66 casos de adenomas hepatocelulares; 14 de neoplasia mucinosa biliar; 13 de hiperplasia nodular focal; 4 de angiomiolipomas; e 3 de hemangiomas. O tamanho médio das lesões foi de 7,6 cm (3,1 a 19,6 cm). A taxa de morbidade total foi de 19%, sendo 9% classificados como Clavien-Dindo 3 ou 4 e não foi observada mortalidade.

**CONCLUSÃO:**

A HVL para tumores hepáticos benignos é segura e apresenta excelentes resultados. No entanto, as indicações para cirurgia são cada vez mais restritas, não sendo recomendável indicar a ressecção somente por se tratar de procedimento minimamente invasivo.

## INTRODUCTION

The laparoscopic approach has gained wide acceptance worldwide, being increasingly employed for the treatment of benign and malignant liver diseases ^7^ ^,^ ^12^. Technical advances and patient outcomes are comparable or even better than open surgery, resulting in the recognition and acceptance of the minimally invasive approach for liver resections. However, it is recommended that laparoscopic liver surgery (LLS) should be performed by a surgeon with experience in liver surgery and with training in advanced laparoscopy, enabling a shallow learning curve. In the early days of the use of LLS, the best candidates were patients with peripheral lesions located in the left lateral and anterior segments, requiring limited resections ^24^. In more recent years, however, an increasing number of major liver resections have been considered safe and feasible when performed in expert centers ^2^ ^,^ ^5^ ^,^ ^10^. Nowadays, even extended resections or transplantations with right lobe living donors are routinely performed by expert groups with excellent results. The advantages of LLS include lower bleeding rates, less postoperative pain, fewer pulmonary complications, shorter recovery time and shorter length of hospital stay, and lower incisional hernia rates ^6^. Since the laparoscopic approach provides excellent surgical results, in addition to improved esthetic outcomes and a prompt return to daily activities, it has become widely accepted for the treatment of benign liver diseases ^14^ ^,^ ^30^.

The majority of laparoscopic liver resections, in its first few years of use, were for benign diseases. In fact, in the first international consensus in Louisville (2008), more than half of all LLS performed up until that time were for benign tumors. At that time, most of the surgeries (20%) were for liver cysts (not precisely liver resections) or for debatable indications, such as focal nodular hyperplasia (FNH) or hemangiomas (36% of the cases) ^24^. Despite LLS being advantageous as a minimally invasive approach, fulfilling the desire of surgeons to perform an easy resection, and enabling easier patient acceptance as a less invasive procedure, it should be emphasized that indications for surgery should be the same as those for open procedures. As the experience with LLS has grown in most groups dedicated to hepatobiliary surgery, and a larger number of patients with malignant diseases underwent surgery, indications for benign tumors have become clearer and more evidence based. In 2016, Ciria et al. reported that 35% of all LLS cases worldwide were for benign diseases ^6^. Similarly, a recent survey including 2,887 patients showed that 43% of the patients underwent surgery due to benign liver diseases ^25^.

This study aims to present our experience with laparoscopic resection of benign liver tumors and to discuss the indications and results from an academic point of view. The authors tried to point out the current indications for resection in an evidence-based context.

## METHODS

All LLS cases from a single referral institution were reviewed from a prospective institutional database (Redcap). Resections for benign tumors of the liver between 2005 and 2021 were then reviewed. Age, sex, size of the nodules, indication for surgical treatment, type of liver resection (minor when up to two segments were resected), necessity of transfusion, morbidity (according to Clavien-Dindo classification) ^9^, length of hospital stay, and mortality were evaluated. Data on continuous variables were collected and set as minimum, maximum, and mean values.

Preoperative diagnosis was based on computed tomography (CT) scans or magnetic resonance imaging (MRI), and in seven cases, a biopsy was necessary. The diagnosis was confirmed via the histological examination of specimen.

## RESULTS

**Figure 1 -:**
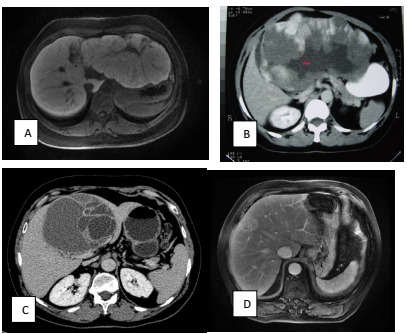
Typical radiological findings for the diagnosis of: symptomatic focal nodular hyperplasia on the left lateral section compressing the stomach (A); symptomatic hepatic hemangioma on the left lateral section compressing the stomach (B); biliary mucinous cystic neoplasm on segment 4 of the liver (C); and AML between segments 4 and 8 (D).

Out of 445 LLS, 100 (22.4%) were for benign tumors, 66 (66%) were for liver cell adenoma (LCA), 14 (14 %) were for biliary mucinous cystic neoplasms (BMCN), 13 (13%) were for FNH, 4 (4%) were for angiomyolipoma (AML) (Figure 1), and 3 (3%) were for liver hemangiomas (LHs). In Table 1, we present the data of LLS for different benign liver tumors on which laparoscopic resection was performed.

**Table 1 -:** Data from patients submitted to laparoscopic resection for benign liver tumors.

		Hepatocellular adenoma	Biliary mucinous cystic neoplasm	Focal nodular hyperplasia	Hepatic hemangioma	Hepatic angiomyolipoma
Number of patients (%)		66 (66)	14 (14)	13 (13)	3 (3)	4 (4)
Sex	Male	9 (13.6)	2 (14.3)	1 (7,7)	1 (33.3)	1 (25)
	Female	57 (86.4	12 (85.7)	12 (92.3)	2 (66.7)	3 (75)
Age (years)	Min-max (mean)	11-70 (35.0)	28-75 (52.5)	16-41 (30.6)	40-69 (56.0)	43-77 (61.75)
Tumor size (cm)	Min-max (mean)	3.1-19.6 (7.0)	4.5-12.5 (7.8)	4.4-14.0 (7.9)	5.0-11.1 (7.6)	5.1-6.4 (5.8)
Length of hospital stay (days)	Min-max (mean)	2-29 (4.8)	1-7 (4.6)	2-9 (3.8)	1-3 (2)	4-18 (8.5)
Extension of resection	Major	12 (18.2)	8 (57.1)	1 (7.7)	0 (0)	1 (25)
	Minor	54 (81.8)	6 (42.9)	12 (92.3)	3 (100)	3 (75)
Operative time	Min-max (mean)	83-495 (253)	120-505 (297)	80-400 (201)	125-210 (157)	120-600 (360)
Estimated blood loss (ml)	Min-max (mean)	10-1,500 (231)	15-1,000 (265)	10-500 (144)	10-20 (15)	50-1,500 (650)
Blood transfusion	Yes	5 (7.5)	0 (0)	0 (0)	0 (0)	1 (75)
	No	61 (92.5)	14 (100)	13 (100)	3 (100)	3 (25)
Perioperative morbidity	No	52 (78.8)	13 (92.9)	11 (84.6)	3 (100)	2 (50)
	Clavien-Dindo grades I/II	7 (10.6)	1 (7.1)	2 (15.4)	0 (0)	0 (0)
	Clavien-Dindo grades III/IV	7 (10.6)	0 (0)	0 (0)	0 (0)	2 (50)

Eighty-six patients were women and 14 were men, with a mean age of 38.6 years (11-77 years). The average sizes of adenomas, BMCNs, FNHs, AMLs, and hepatic hemangiomas were 7.0, 7.8, 7.9, 5.8, and 7.6 cm, respectively. In 22.4% of cases, major HVL LLSwas performed. The estimated blood loss was 245 ml (10-1,500 ml), and the transfusion rate was 6%. The morbidity of the series was 19.3%, but only 9.1% had complications and classified as Clavien-Dindo grades 3 or 4.

The most common complications were pneumonia (three cases), intra-abdominal abscess (two cases), and biliary fistulas (two cases). The average length of hospital stay was 4.6 days. No mortality was observed. Preoperative diagnostic error was observed in nine cases; however, all of them were confirmed positive for FNH via histological examination performed postoperatively. In these cases, the preoperative hypotheses for resection were liver adenoma in eight cases and hepatocellular carcinoma in one case.

## DISCUSSION

The improvements in diagnostic tools during the last few years led to an increase in the detection of benign liver tumors. In addition, more accurate diagnoses became possible, allowing liver surgeons to understand the behavior of these benign lesions. The increase in diagnosis, associated with enthusiasm for the minimally invasive technique, led to an increase in indications for surgery. Toro et al. have shown that the introduction of the laparoscopic approach resulted in an increasing rate of liver resections for benign tumors, even in those with doubtful indications ^28^.

Indications for the resection of benign liver tumors are observed as follows: (1) symptomatic diseases that impact the quality of life and/or (2) risk of complications, such as malignant transformation or rupture. The most frequent indications for LLS are LCA and BMCN due to the risk of complications. The indications for the resection of hemangiomas or FNH are anecdotal cases where significant symptoms are present. In this study, we discussed each of these tumors, focusing on the clinical indications for resection.

There is a consensus that the indications for the laparoscopic resection of benign liver tumors, despite the procedure being feasible and safe, should not be expanded in the face of the adoption of a lesser invasive approach ^10^ ^,^ ^23^ ^,^ ^24^. Low morbidity rates (<15-20%) and no mortality ^12^ ^,^ ^13^ ^,^ ^29^ are the goals of post-LLS.

In our experience with 100 laparoscopic resections for benign liver tumors, the main indication was LCA (n=66; 66%). In the whole series, there were 22 (22%) major liver resections, and the mean size of the lesions was 7.6 cm. The estimated blood loss was 245 ml, 6.0% of the patients received a blood transfusion, and no conversions to open surgery were necessary. Significant postoperative complications (Clavien-Dindo ≥3) were observed in 9.0% of the patients, with no operative mortality. The mean length of hospital stay was 4.6 days (Table 1).

Each of the indications for the resection of benign liver tumors is discussed below.

### Liver cell adenoma

The LCA is a rare benign liver tumor that affects young women of childbearing age, with an increasing incidence due to the widespread use of oral contraceptives (OCs) ^3^ ^,^ ^15^ ^,^ ^20^. LCA is most often asymptomatic and is frequently found incidentally during radiological imaging for unrelated causes. The estimated incidence of LCA is one per one million inhabitants, rising to 30-40 per million in long-term OC users ^20^. The association with glycogen storage disease types 1 and 3, the use of anabolic steroids, obesity, and metabolic syndromes is also well-established ^20^.

Based on the genetic and phenotypic characteristics, four LCA subgroups have been reported initially: (a) hepatocyte nuclear factor 1-alpha (HNF1α)-mutated LCA, representing 30-40% of LCA cases, is characterized by inactivating mutations in the gene HNF1α and leads to a fatty phenotype nodule and negative liver fatty acid-binding protein expression, also known as “steatotic adenoma”; (b) inflammatory LCA (I-LCA), representing 40-50% of all adenomas, has inflammatory characteristics related to JAK-STAT pathway activation and is morphologically characterized by telangiectasias, inflammation, ductular reaction, and staining for C-reactive protein; (c) β-catenin-mutated LCA (β-LCA), representing 10% of LCAs, has variable morphologies, positive glutamine synthetase staining, and nuclear expression of β-catenin; and (d) approximately 10% of adenomas remained unclassified, with no characteristic histological features ^21^.

More recently, a new LCA molecular classification was proposed based on the molecular profile in four different pathways: activation of β-catenin, interleukin 6/JAK/STAT, the sonic hedgehog pathway, and HNF1α inactivation. Based on these molecular features, a new subtype was identified (the sonic hedgehog LCA [shLCA]), and the β-LCAs were divided into two further subgroups according to the presence of CTNNB1 mutations (exon 3 vs. exons 7/8). According to this new classification, the presence of CTNNB1 mutations on exon 3 is associated with a high risk of malignant transformation. In addition, the shLCA was associated with a high risk of rupture and bleeding. Notably, some tumors present mixed features common to inflammatory and β-catenin-activated subtypes (mixed b^exon7/8^I-LCA and b^exon3^I-LCA). In this new classification, 7% of LCAs still remain unclassified ^21^ ^,^ ^22^.

The new classification with eight LCA subtypes shows the complexity and heterogeneity of the disease. However, from a practical point of view, it is not useful since a complex molecular process to identify all subtypes is necessary. Moreover, the whole adenoma specimen is needed for precise evaluation. For these reasons, this classification is not yet fully applicable in daily practice ^20^.

The new knowledge of the molecular profile and its impact on clinical behavior led to changes in the diagnosis and treatment of this challenging disease. In daily practice, the diagnosis of LCA subtypes is based on MRI findings whose capacity to distinguish different LCA subtypes (especially steatotic and inflammatory HA) is well-established ^29^. In cases of doubt, a percutaneous biopsy can be performed.

The changes in treatment strategy are ongoing, based on the risk of complications according to the adenoma subtypes. Until recently, in women with adenomas measuring 5.0 cm or more, resection was indicated due to the estimated risk of 5-8% for malignant transformation or 21-29% for rupture and hemorrhage. Nault et al. have reported a large series with 411 patients, showing that symptomatic bleeding occurred in 14% of the patients and that 3% of the patients presented malignant degeneration from LCA to hepatocellular carcinoma ^23^. Farges et al. found that the prevalence of malignancy in patients with LCA was 10 times higher in men than in women, suggesting the routine resection of all LCAs in men, irrespective of lesion size ^12^. Currently, resection should be recommended for all adenomas affecting men and for nonsteatotic adenomas larger than 5 cm, after 6-12 months of OC interruption and weight loss, in women ^14^.

When surgical treatment is indicated, LLS was found to provide excellent results, even when major resections were required ^20^. We have previously shown excellent results following LLS for the treatment of LCA, with a low rate of complications and no mortality ^13^. Patients with LCA seem to be especially benefited from the laparoscopic approach, in particular, young female patients, in which a less invasive approach offers better postoperative outcomes and excellent cosmetic results. The laparoscopic approach should be considered the standard of care for patients with LCAs when performed by an expert surgeon ^13^ ^,^ ^14^.

In our experience, 66 patients with LCA underwent laparoscopic resection, including 12 (18.2%) major liver resections, with low morbidity rates (10.6% classified as Clavien-Dindo grades 3 or 4) and no mortality. The open approach was employed for the resection of centrally located lesions or in patients with very large tumors (>10 cm).

### Biliary mucinous cystic neoplasm

The BMCN is a rare neoplastic cyst that was originally called biliary cystadenoma. These premalignant cystic tumors are often detected incidentally on radiological imaging for other causes ^27^. These lesions originate from the biliary epithelium and represent <1% of all liver cystic lesions ^11^. Edmondson described BMCN as a multilocular cystic lesion lined by columnar epithelium with an accompanying dense cellular “ovarian-like” stroma ^1^.

BMCNs are typically detected in young women (>90%) between 30 and 50 years of age and are rarely observed in men. BMCNs are usually large complex cysts often located in the left lobe of the liver with multiple septations, and papillary projections may be seen originating from the septa or the cyst wall. The frequency of malignant degeneration has been reported in the range between 20% and 30% of these cases ^1^. For this reason, resection is the treatment of choice, offering the best chance of a cure, and both open and minimally invasive approaches can be employed, depending on the size, location of the lesion, and the proximity to biliary and vascular structures. Enucleation of these lesions can be a very complex procedure once BMCNs are situated very close to the biliary duct (left duct or biliary confluence) and, oftentimes, a left hepatectomy is the procedure of choice.

In the largest series to date reporting 221 cases, the 5-year recurrence-free survival was 61.4%, and recurrences tended to occur locally, often caused by an incomplete initial resection ^18^. In our series, we treated 14 patients with BMCN, all situated in the left lobe of the liver and most being large lesions (mean size=7.8 cm; range=4.5-12.5 cm). Eight left hepatectomies and six cystectomies were performed with no Clavien-Dindo grades 3 or 4 morbidity and no mortality. No recurrence was observed.

### Focal nodular hyperplasia

The FNH) is the second most common benign liver tumor, generally diagnosed incidentally during an abdominal ultrasound. FNH usually affects women between 30 and 50 years of age, and its incidence is not influenced by the use of OCs. It is characterized by the presence of a well-delineated hypervascular mass with fibrous septa and a central stellate nonenhancing scar. FNH represents a hyperplastic response to an arteriovenous malformation, being more common in young women, although up to 10% of cases may be in men ^15^. It has a benign clinical course, and most cases are asymptomatic; mild abdominal discomfort is rarely observed. Since FNH rarely has complications, with no risk of rupture or malignant degeneration, treatment is seldom required ^17^.

There are rare situations where resection can be indicated, such as a large left-sided mass leading to symptomatic gastric compression or a rapid growth lesion.

In our experience, we resected 13 FNHs, with 8 misdiagnosed as LCAs and 1 as hepatocellular carcinoma (9 misdiagnoses); one patient underwent surgery due to rapid tumor growth and another three due to a large left lateral segment tumor leading to gastric compression symptoms (Figure 1).

### Liver hemangioma

The LH is the most common benign liver tumor with an unknown etiology and with a female preponderance (5:1 female-to-male ratio) ^16^. This congenital vascular malformation composed of a tangle of blood vessels may present estrogen receptors.

Most patients with LH are asymptomatic, and hemangioma is usually an incidental finding on nonrelated radiological evaluation. Large hemangiomas may eventually cause abdominal pain due to intratumoral thrombosis. LH resection is rarely necessary except in the following circumstances: symptoms with a poor response to painkillers, an impact on the patient’s quality of life, the Kasabach-Merrit syndrome, and a large left-sided tumor with symptomatic gastric compression. Complications, including hemorrhage or rupture, are extremely rare. The rare Kasabach-Merritt syndrome occurs in <3% of the patients and is caused by the trapping of platelets within the tumor, leading to an activation of the clotting cascade, resulting in thrombocytopenia and fibrinolysis.

A large series from our group retrospectively evaluated 249 patients with hemangiomas, of which 27.3% were larger than 4 cm and 6.4% were larger than 10 cm. Notably, 30% of patients were symptomatic; however, only eight patients (3.2%) underwent surgical treatment ^16^.

In our series, a conservative nonsurgical approach was always adopted; considering that although complications might be prevalent, they are extremely rare. Resection should be avoided even in the presence of pain because liver resection presents higher morbidity rates when compared with the natural course of the disease. Other etiologies for pain (i.e., dyspeptic syndrome, gallbladder disease) should be investigated, and if LH is determined to be the cause of pain, it should be controlled with painkillers. The enucleation of the hemangioma when surgery is indicated is feasible; however, in our experience, it leads to more bleeding; thus, we preferred to cut through the normal liver tissue close to the tumor to preserve the parenchyma. Special attention should be given to patients who have hemangiomas larger than 10 cm; in these cases, significant pain is more prevalent (37.5%), but the size of the lesion on its own should not be an indication for resection. In these large tumors, laparoscopic resection is quite tricky and, sometimes, impossible due to the difficult mobilization of the liver. In our experience, the median size of resected LHs was 7.6 cm, and the main indications were abdominal pain (one case) and large left lateral segment tumors leading to gastric compression (two cases) (Figure 1). In these very few cases in which resection was performed, no morbidity or mortality was observed.

### Angiomyolipoma

The AML is a rare solid mesenchymal tumor, usually affecting the kidneys, being part of the group of perivascular epithelioid cell tumors. Hepatic AML is rare, with approximately 600 reported cases ^19^. In CT- or MRI-contrasted evaluation, it appears as a hypervascular tumor with a washout phase mimicking hepatocellular carcinoma arising in normal liver parenchyma ^26^, representing a diagnostic challenge, especially when the fat content at radiological evaluation is low (Figure 1).

Hepatic AML usually presents a benign course. However, an aggressive behavior with recurrent disease or metastasis can be observed, although there are no data at the time of writing to predict the natural course of this tumor.

Due to its difficult diagnosis and an eventual potentially aggressive behavior, a biopsy to confirm the diagnosis and evaluate cellularity might be necessary ^19^.

The criteria to evaluate ALM behavior were recently proposed ^4^:

- Benign (no worrisome features): tumor size <5 cm, well-delineated with no infiltration, low nuclear grade and cellularity, mitotic activity ≤1/50 HPF, and no vascular invasion.
- Uncertain malignant potential: pleomorphism/multinucleated giant cells only or size >5 cm.
- Aggressive behavior: two or more of the following worrisome features: size >5 cm, peripheral infiltration, high nuclear grade and cellularity, mitotic activity >1/50 HPF, and vascular invasion.

According to the World Health Organization classification of tumors, the main risk predictors of a more aggressive disease and metastatic behavior are significant nuclear atypia, diffuse pleomorphism, and mitotic activity >1 mitosis per mm². Therefore, in the presence of large tumors, or when biopsy depicts pleomorphism or mitotic activity, resection is recommended.

Finally, in our series, four patients with hepatic AML underwent resection. These cases presented tumors larger than 5 cm and underwent percutaneous biopsy to confirm the diagnosis. These patients underwent LLS due to an uncertain malignant potential with excellent outcomes and no recurrence.

## CONCLUSIONS

The LLS for benign liver tumors is feasible and safe, even when major resections are required. Moreover, less pain, a shorter recovery time, and better cosmetics are the norms. Each benign liver tumor has a specific and restrictive indication for resection. These indications are usually due to the presence of symptoms that impact the quality of life or the risk of complications, such as malignant degeneration or rupture and bleeding. Before considering the surgical indication, a multidisciplinary case discussion is recommended not only to confirm the diagnostic hypothesis or the eventual need for a biopsy but also to establish the best therapeutic approach.

The authors reemphasized that indications for resection should be the same as those for open surgery and that the range of indications should not be widened just because it is a minimally invasive procedure. In the few cases where resection is necessary to treat benign liver tumors, laparoscopic liver resection should be the preferred method.
